# Visualizing catalyst heterogeneity by a  multifrequential oscillating reaction

**DOI:** 10.1038/s41467-018-03007-3

**Published:** 2018-02-09

**Authors:** Yuri Suchorski, Martin Datler, Ivan Bespalov, Johannes Zeininger, Michael Stöger-Pollach, Johannes Bernardi, Henrik Grönbeck, Günther Rupprechter

**Affiliations:** 1 Institute of Materials Chemistry, Technische Universität Wien, 1060 Vienna, Austria; 2 USTEM, Technische Universität Wien, 1040 Vienna, Austria; 3 Department of Physics and Competence Centre for Catalysis, Chalmers University of Technology, SE-412 96 Gothenburg, Sweden

## Abstract

It is well documented that different surface structures of catalytically active metals may exhibit different catalytic properties. This is typically examined by comparing the catalytic activities and/or selectivities of various well-defined smooth and stepped/kinked single crystal surfaces. Here we report the direct observation of the heterogeneity of active polycrystalline surfaces under reaction conditions, which is manifested by multifrequential oscillations during hydrogen oxidation over rhodium, imaged in situ by photoemission electron microscopy. Each specific surface structure, i.e. the crystallographically different µm-sized domains of rhodium, exhibits an individual spiral pattern and oscillation frequency, despite the global diffusional coupling of the surface reaction. This reaction behavior is attributed to the ability of stepped surfaces of high-Miller-index domains to facilitate the formation of subsurface oxygen, serving as feedback mechanism of the observed oscillations. The current experimental findings, backed by microkinetic modeling, may open an alternative approach towards addressing the structure-sensitivity of heterogeneous surfaces.

## Abstract (web summary)

The structurally different domains of a polycrystalline material may exhibit differing catalytic properties. Here, the authors directly visualize this phenomenon by observing the catalytic hydrogen oxidation that oscillates, simultaneously exhibiting different frequencies for structurally different rhodium domains.

## Introduction

Self-sustained oscillations, i.e. oscillations occurring without an external stimulus, are a fascinating phenomenon in nature, originating from self-organization in a wide range of fields, including biology, chemistry, physics, sociology, and even economics. In simple words, a certain property or parameter changes periodically, despite that external conditions are constant. Examples of such oscillations range from fluid mechanics^1^ via ecosystems (described, e.g., by classic Lotka−Volterra predator−prey models^2^) to real estate markets^3^. In chemistry, more than a hundred chemical reactions are known to exhibit oscillating reaction kinetics under stationary conditions, the most famous being the Belousov−Zhabotinski^4^ and Bray−Liebhafski^5^ (chemical clock) reactions.

In the field of heterogeneous catalysis, when e.g. gaseous reactants react on solid catalytically active surfaces, oscillations were first reported in the 1970s for CO oxidation on Pt^6^, and in the 1980s for NO reduction^7^: the product formation rate measured by mass spectroscopy or gas chromatography varied periodically, despite constant external conditions (gas pressures, temperature, flow). Since then, oscillatory surface reactions have developed into a wide research field^8,9^ having also practical impact: non-steady, e.g. periodically oscillating, operation regimes may improve catalytic reactor performance^10^ or enable fine tuning of surface activity^11^. Apart from rate oscillations, many types of spatial−temporal self-organization of initially uniformly distributed reactants were observed, ranging from concentric self-repeating patterns to chaotic behavior^8,9,12^.

To date, the main body of work on oscillating surface reactions is still related to CO oxidation^8,13^ and NO reduction^14,15^, mainly on single crystals of Pt group metals (Pt, Pd, Rh, which are widely employed in automotive catalytic converters). The mechanism of oscillating CO oxidation was thoroughly studied by Ertl and co-workers by application of surface analysis techniques to well-defined homogeneous low-Miller index single crystal surfaces^8,9^. The importance of self-organization phenomena and oscillating surface reactions was reflected by the Nobel Prizes to Prigogine in 1977 and to Ertl in 2007.

Despite the increasing importance of hydrogen-based energy generation in fuel cells^16^, much less attention has been given to (oscillating) catalytic H_2_ oxidation. There are only few reports on oscillating H_2_ oxidation on polycrystalline Pt and Pd wires^17,18^, polycrystalline Pt layers^19^, or supported Pd and Rh catalysts^20,21^ under atmospheric pressure conditions. Under high vacuum conditions, enabling microscopic studies of H_2_ oxidation, oscillations have been observed only on well-ordered bimetallic Rh/Ni surface alloys^22^ and on sharp Rh nanotips under high electric fields (>10 V/nm; with the applied field causing the oscillations by stimulating the periodic formation of Rh surface oxide^23^). However, from a technological point of view, H_2_ oxidation studied under field-free conditions over less-ideal heterogeneous surfaces exhibiting various surface structures as well as steps, kinks and defects, may be more relevant.

In this work, we report the direct observation of multifrequential oscillations occurring during the hydrogen oxidation reaction over a heterogeneous rhodium surface composed of µm-sized domains of different crystallographic orientations. In situ monitoring of the reaction by photoemission electron microscopy (PEEM) reveals that each particular domain exhibits its own local oscillation frequency which appears to be governed by the local Rh surface structure, with structure-sensitive subsurface oxygen formation serving as feedback mechanism, as rationalized by a micro-kinetic model.

## Results

### Multifrequential oscillations on individual domains of a polycrystalline Rh foil

**Fig. 1:**
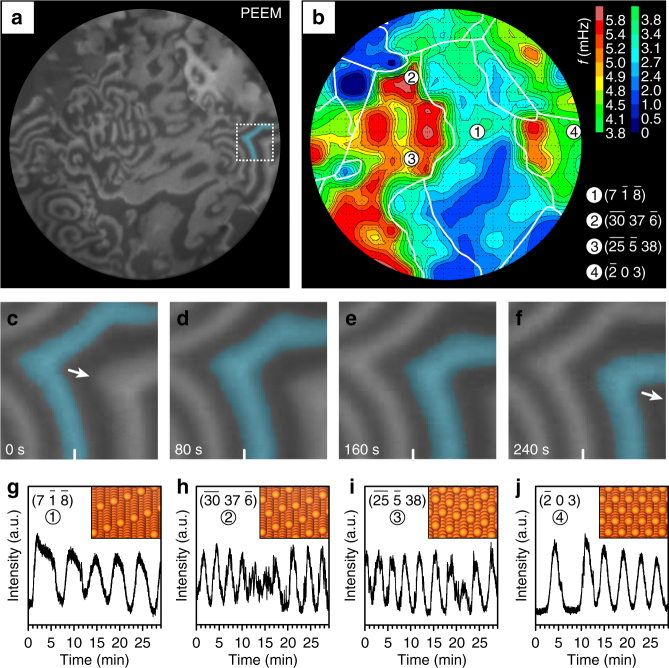
Isothermal kinetic oscillations in H_2_ oxidation on polycrystalline Rh on a µm scale. (**a**) PEEM snapshot (field of view 520 µm) taken during H_2_ oxidation at constant *p*_O2_ = 1.1 × 10^−6^ mbar, *p*_H2_ = 8.4 × 10^−7^ mbar, and T = 433 K; (**b**) “frequency map” of the observed oscillations. Crystallographically different domains are marked with white lines (see also Supplementary Note 1). The numbered circular symbols mark the selected crystallographic orientations; (**c**-**f**) propagation of a chemical wave in the 70 × 70 µm^2^ section marked in (**a**); (**g**–**j**) time-dependent (oscillating) local PEEM intensities of selected regions. The positions of the corresponding circular ROIs (of 1 µm diameter) are placed in the centers of the circular symbols in (**b**). The ball model insets illustrate the stepped surface structure of the selected regions, the Miller indices of which were determined by EBSD measurements (see Supplementary Note 1)

For the current study, we have used PEEM to visualize in situ the ongoing H_2_ oxidation on polycrystalline Rh foil, which consisted of differently oriented stepped (high Miller-index) domains of 10–50 µm size (Fig. 1). The crystallographic orientation and the chemical purity of the individual domains were characterized by EBSD (electron backscatter diffraction; grain boundaries are marked as white lines in Fig. 1b) and XPS (X-ray photoelectron spectroscopy), respectively. Accordingly, the Rh foil represented a well-defined mesoscopic model catalyst with known size, shape, and crystallographic orientation of each individual domain. The PEEM chamber was used as a flow reactor for catalytic H_2_ oxidation, with precision leak valves for reactant dosing^8–13,22,24^.

Figure 1a displays a PEEM video-frame during H_2_ oxidation on polycrystalline Rh foil, at constant *p*_O2_ = 1.1 × 10^−6^ mbar, *p*_H2_ = 8.4 × 10^−7^ mbar, and constant *T* = 433 K. The dark areas correspond to the oxygen-covered inactive surface, and bright zones to the catalytically active oxygen-free surface with low hydrogen coverage^24^. In situ PEEM videos (see Supplementary Note 2 and Supplementary Movie 1) reveal, however, not a static picture, but a complex turbulence-like “stirring” surface consisting of repeatedly nucleating “spirals” which spread as chemical waves (magnified view in Fig. 1c–f; one wave has been colored). The spirals overlap at the domain boundaries (Fig. 1b) and create the turbulence-like pictures (Fig. 1a). It is most intriguing that the “rotation speed” of the spirals strongly varies for different surface orientations.

Placing regions of interest (ROIs, each corresponding to a 1-µm-wide circle on the surface) at different positions of the image (Fig. 1b), specifically at different crystallographic domains, enables to evaluate the local image brightness, which reflects the local reaction rates (kinetics by imaging^25^). The image brightness analysis, thus, reveals the local oscillation frequencies, as illustrated in Fig. 1g–j. Since the crystallographic orientation of the individual domains is known from EBSD (see labels in Fig. 1b), the local oscillation frequencies can be correlated with the local surface structure of the corresponding domains. The observed local oscillation frequencies can then be displayed as a “frequency map”, shown in Fig. 1b.

### Local oscillations confined in a furrow-like defect on Rh(111)

**Fig. 2:**
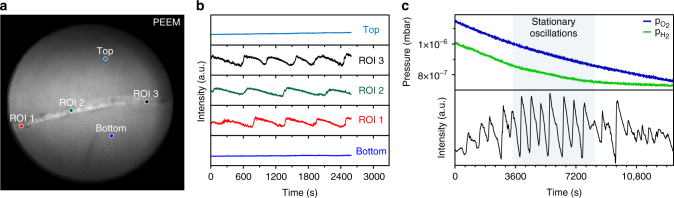
Oscillations in H_2_ oxidation confined within a furrow-like defect on a Rh(111) surface. (**a**) PEEM image (field of view 520 µm) of ongoing H_2_ oxidation, with the smooth Rh(111) surface being in the active steady state, whereas pulse-like oscillations occurred inside the furrow; (**b**) local PEEM intensity registered on the smooth surface (“top” and “bottom”) and within the defect (ROIs 1−3); (**c**) existence diagram for oscillations in ROI 3 (the blue and green curves show the partial pressures for O_2_ and H_2_, respectively, the temperature was constant at 433 K)

It was not possible to induce oscillations on smooth low-index single crystal surfaces of Rh under these (and also under significantly varying *p*_O2_, *p*_H2_ and *T*) conditions. It seems that a certain degree of surface roughness is required to generate oscillations. To prove this assumption, H_2_ oxidation was examined on a smooth Rh(111) single crystal surface containing a mesoscopic furrow-like defect (25–30 µm wide, 1 µm deep; a “scratch”) (Fig. 2a). The result is shown in Fig. 2b, in which the time dependencies of the local PEEM intensities are shown for different ROIs (inside and outside the furrow), and in Fig. 2c, displaying an oscillation existence diagram. Clearly, the oscillations occur solely within the furrow and the confinement of the oscillating behavior to the µm-sized defect on the otherwise smooth Rh surface corroborates the importance of steps and kinks for the generation of oscillations. It has been reported that the initiation of kinetic transitions is pinned to surface defects^26,27^; the present study suggests, however, that not only the initiation, but also the propagation of oscillating fronts in H_2_ oxidation on Rh need a sufficiently rough surface. The analysis of PEEM video-frames confirms the “propagating wave” character of the observed instabilities confined within the furrow (see Supplementary Figure 3). Further analysis of the furrow-profile by AMF (atomic force microscopy) and EBSD (see Supplementary Note 1) demonstrated that the steep flanks of the furrow consisted of highly stepped surfaces, very different from the surrounding smooth Rh(111) surface. This explains why self-sustained oscillations of hydrogen oxidation have not yet been observed for low-index (quasi step-free) single crystal planes of Pt group metals.

Returning to Fig. 1g–j, the width of the step-terraces apparently influenced the oscillation frequency and, last but not least, also the shape of the steps had an effect (cf. Fig. 1i, j). Note that small variations of the oscillation frequency within one domain may result from diffusional coupling of neighboring regions or small local surface structure deviations (cf. Supplementary Note 1, Supplementary Figure 1d).

### Feedback mechanism and micro-kinetic model calculations

Generally, a surface reaction can only exhibit oscillating behavior when a feedback mechanism exists beside the bistability^9^. Bistability describes the existence of two alternative steady states (active or inactive in the present case) under identical external conditions; thus the system’s state is determined by its previous history. The feedback mechanism, in turn, periodically switches between these two steady states, e.g. by varying the sticking coefficient of reactants via changing the surface structure^8,9^, by changing the concentration of subsurface oxygen^8,28^ or by (surface) oxide formation^12,28^. The essential role of the stepped Rh surface in the present observations, and the fact that oscillating H_2_ oxidation was not observed on smooth low index Rh surfaces (neither in the present study nor earlier), suggests the periodical formation and depletion of subsurface oxygen to be the feedback mechanism in the present observations. An alternative feedback mechanism based on surface reconstruction, known from oscillating CO oxidation on some Pd and Pt single crystal surfaces^8,9,13,15^, is highly unlikely here, since it can hardly be expected that dozens of differently oriented Rh domains would simultaneously exhibit similar reconstructions, but with different frequencies.

**Fig. 3:**
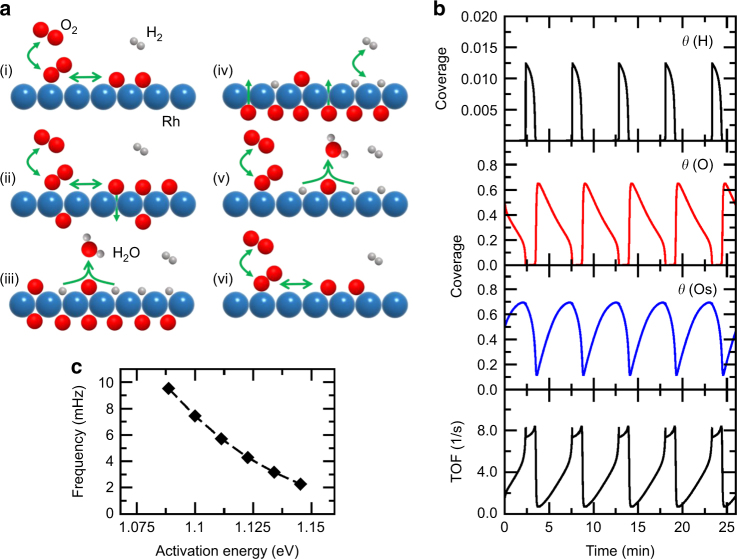
Reaction steps and micro-kinetic model calculations. (**a**) Schematic representation of the reaction steps in H_2_ oxidation on Rh. Color code: Rh (blue), O (red), and H (gray). (**b**) Top to bottom panels show the coverage of hydrogen (H), of oxygen (O), of subsurface oxygen (Os) and the reaction rate. (**c**) Oscillation frequency as a function of the activation energy for oxide formation. The simulations have been performed at *T* = 433 K and partial pressures of oxygen and hydrogen of 1.1 × 10^−6^ mbar and 8.4 × 10^−7^ mbar, respectively

Figure 3a illustrates the main reaction steps in the oscillating cycle: (i) upon competitive coadsorption, oxygen from the mixed O_2_/H_2_ gas phase adsorbs dissociatively on the Rh surface via a molecular precursor; (ii) atomic oxygen can then diffuse from surface to subsurface sites and subsequently form a surface oxide layer. Both subsurface O and surface oxide significantly lower the sticking probability of oxygen and thus reduce the rate of oxygen adsorption; as a result, (iii) hydrogen can now also adsorb dissociatively, despite the competitive adsorption with oxygen, and water is formed by reaction between adsorbed hydrogen and oxygen; (iv) upon continuing reaction, subsurface oxygen is slowly depleted as it diffuses to the surface and is consumed in the reaction with hydrogen; (v) when the concentration of subsurface oxygen becomes low enough, the sticking coefficient of oxygen and thus the high rate of oxygen adsorption recover and the surface switches back to the inactive state of high oxygen coverage, (vi) closing the oscillation cycle.

The reaction rate minima/maxima during oscillations are, thus, characterized by the high/low oxygen surface coverage, which can be unambiguously identified by PEEM: the low-activity state with high oxygen coverage induces the dark image contrast, whereas the high-activity regions with low oxygen coverage (surface covered by hydrogen) are characterized by bright contrast.

The present PEEM observations do not provide a direct evidence for the formation of subsurface oxygen but its formation has been directly observed^29^ for the smooth Rh(111) surface at 470 K. For the rougher Rh surfaces studied herein, subsurface oxygen might form at slightly lower temperature. To rationalize the suggested model and, particularly, to verify the feedback mechanism, the observed oscillations were analyzed by a mean-field micro-kinetic model (Supplementary Note 3). The kinetic parameters were based on the values for low index Rh surfaces used in refs. ^30,31^ to explain the field-induced oscillations in H_2_ oxidation on Rh nanotips.

The model predicts a critical dependence of oscillations on the feedback loop between the rate of oxygen adsorption and the concentration of subsurface oxygen. At the current experimental conditions, on Rh(111) the rate of formation of subsurface oxygen and surface oxide is too low, which prevents oscillating reaction kinetics to be established. Thus, in agreement with the experiments, the micro-kinetic model does not predict oscillations to occur on a smooth Rh(111) surface. For the stepped Rh surfaces under the current experimental conditions, the exact kinetic parameters are unknown and difficult to obtain with sufficient accuracy from first-principles calculations. However, we noticed that the activation energies for surface oxidation and surface reduction calculated by density functional theory for Rh(111), Rh(011), and Rh(001) correlate linearly. Thus, as a pragmatic procedure, we reduced the activation energy for oxidation (as expected for stepped Rh surfaces for which the incorporation of oxygen atoms occurs easier), and scaled (keeping the other parameters fixed) the activation energy for reduction accordingly, until the (field-free) oscillations occurred in the simulations. In this way, reaction kinetics were obtained that mimic the experimentally observed oscillating behavior (Fig. 3b).

The frequency of oscillations sensitively depends on the rate of subsurface oxygen formation and its depletion, both governed by the activation energy of subsurface oxygen formation. Reducing the activation energy from the value used in Fig. 3b (1.134 eV) by only 2% increases the frequency by a factor of two (Fig. 3c). The more easy the subsurface oxygen is formed, the higher the predicted oscillation frequency is, which is indeed observed in the experiments: the more “stepped” and “kinked” the surfaces were (Fig.1b), the higher the oscillation frequency was, and the faster the spirals rotated. This model also explains the observations of field-induced oscillations on Rh nanotips^30,31^: as shown earlier^32^, a high electric field lowers the activation energy for surface oxidation of Rh, enabling oscillations under high vacuum conditions.

## Discussion

In summary, multifrequential oscillating spatio-temporal patterns, formed by spreading chemical waves, were observed by PEEM during the ongoing H_2_ oxidation on µm-sized stepped high-Miller-index domains of polycrystalline Rh. These spirally shaped chemical waves, generated by local surface defects, spread across the grain boundaries which act as “frequency transformers” from one domain to another. As a result, the local reaction rate oscillates with an individual frequency which is associated with the local surface structure of each particular domain. The microkinetic model explains the correlation of the local oscillation frequency and the local surface structure: the frequency of the observed oscillations is governed by the feedback mechanism, in the present case the subsurface oxygen formation, whose rate is structure-sensitive. The critical sensitivity of the oscillation frequency on the activation energy of subsurface oxygen formation explains why oscillations in H_2_ oxidation were not observed on smooth Rh surfaces despite decades-long efforts: the absence of steps, kinks and other low coordinated sites makes the formation of subsurface oxygen difficult. The feedback mechanism “stutters” and oscillations can thus not be established. In contrast, on stepped and kinked Rh surfaces the more facile formation of subsurface oxygen enables self-sustained oscillations, which allow one to directly visualize the catalytic heterogeneity of surfaces active in hydrogen oxidation. This may open a promising approach towards addressing the structure-sensitivity of heterogeneous surfaces and adds to the fundamental understanding of complex self-organized systems.

## Methods

### Visualization of H_2_ oxidation on Rh

The current experiments were performed in a PEEM/XPS ultrahigh vacuum setup consisting of separate PEEM and XPS chambers connected with each other by a sample transfer. The setup is equipped with a PEEM (Staib Instruments), a deuterium discharge UV lamp (photon energy ~6.5 eV) for electron excitation, an XPS-system (Phoibos-100 hemispherical energy analyzer and XR 50 twin anode X-ray source, both from SPECS), a high purity gas supply system (O_2_: 99.99%, H_2_: 99.97%) and sample preparation facilities for cleaning the sample by argon ion sputtering and subsequent annealing. The reaction was visualized in situ by PEEM and the images were recorded by a CCD camera (Hamamatsu). The PEEM magnification was calibrated by comparison of PEEM images of the Rh samples with optical micrographs of the same Rh foil and of the same Rh(111) single crystal.

### Preparation and characterization of Rh samples

The polycrystalline Rh sample consisted of a 10 × 12 mm^2^ polished polycrystalline Rh foil of 0.2 mm thickness (Mateck, purity 99.99%) which was cleaned in UHV by repeated cycles of sputtering with Ar^+^ ions at 1 keV at 300 K and consecutive annealing to 973–1073 K. The cleanness of the sample was confirmed by XPS before each single reaction measurement. The sample temperature was measured by a Ni/NiCr thermocouple spot-welded directly to the sample. The crystallographic orientation of individual μm-sized domains of a polycrystalline Rh foil was determined by EBSD (Electron Back Scattering Diffraction). The EBSD measurements were performed by a field emission scanning electron microscope (FEI Quanta 200F) using standard EBSD conditions and evaluation procedures. Further details on the EBSD characterization can be found in Supplementary Note 1. The Rh(111) single crystal (Mateck, purity 99.99%) of 10 × 10 mm^2^ size and 0.6 mm thickness was cleaned in a similar Ar^+^ sputtering and annealing procedure as the Rh foil with a subsequent XPS control.

### The micro-kinetic model

The micro-kinetic model is based on the well-established Langmuir−Hinshelwood mechanism for H_2_ oxidation on Rh, with the reaction network including the dissociative adsorption and associative desorption of hydrogen, dissociative adsorption (and associative desorption) of oxygen via a precursor state, formation and reduction of subsurface oxygen and catalytic water formation. The details of the model and of the calculations as well as the used calculation parameters are presented in Supplementary Note 3.

### Data availability

The data that support the findings of this study are available from the corresponding author upon request.

## Electronic supplementary material

Supplementary Information

Description of Additional Supplementary Files

Supplementary Movie 1
