# Supplementary material for: Visualizing catalyst heterogeneity by a multifrequential oscillating reaction
Source: Nat Commun. 2018 Feb 9;9:600. doi: 10.1038/s41467-018-03007-3 (PMC5807506; doi:10.1038/s41467-018-03007-3)
Supplement: Supplementary file 1 — Supplementary Information [file 41467_2018_3007_MOESM1_ESM.pdf]

Visualizing catalyst heterogeneity by a multifrequential oscillating reaction

Suchorski et al.

Supplementary Note 1: EBSD characterization of Rh samples

Individual domains of the polycrystalline Rh foil

To study processes on individual µm-sized domains of a polycrystalline metallic foil, their exact crystallographic orientations must be determined first. In the present study, this was carried out by EBSD (Electron Back Scattering Diffraction). The EBSD measurements were performed by a field emission scanning electron microscope (FEI Quanta 200F) using standard EBSD conditions and evaluation procedures [1]. A corresponding result is shown in Supplementary Figure 1b, in which the same field of view as that in Supplementary Figure 1a (the PEEM image of the Rh foil) is shown, but with the crystallographic orientations marked. Four examples of cristallographically different stepped Rh surfaces are illustrated by ball models in Supplementary Figure 1c. A region of deviating crystallographic orientation within the $(\overline{30}\,37\,\overline{6})$ facet is indicated in Supplementary Figure 1b and is shown in a magnified view in Supplementary Figure 1d.

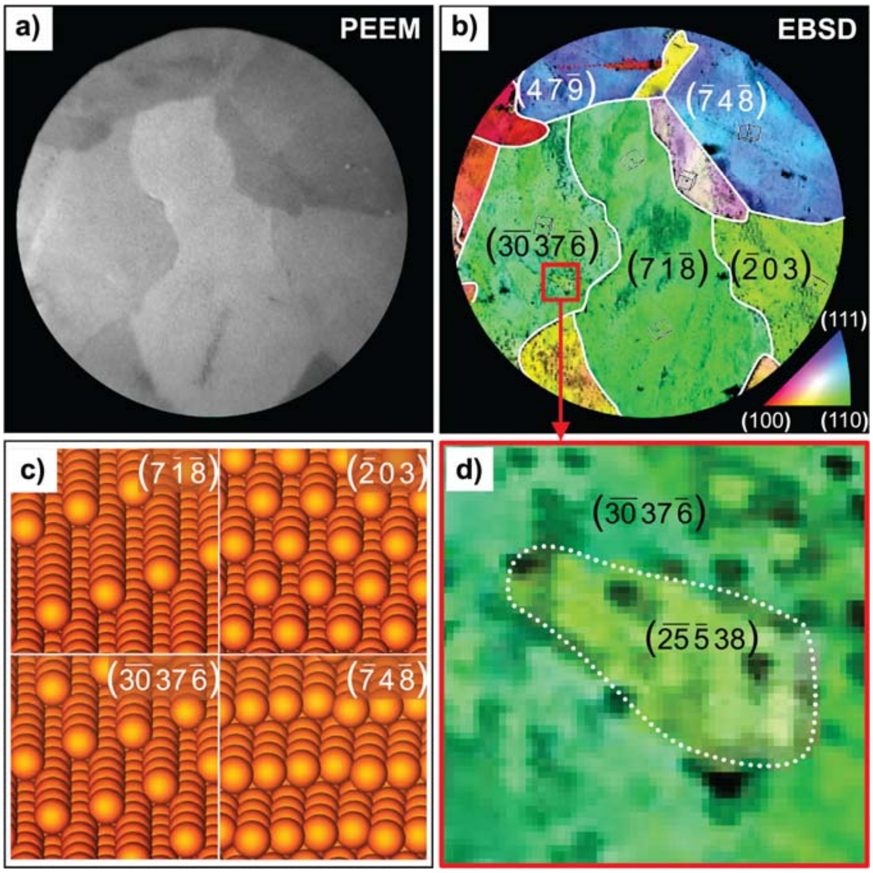

Supplementary Figure 1. Determination of the crystallographic orientation of individual domains of a polycrystalline Rh foil: a) PEEM image of a clean Rh foil consisting of µm-sized differently oriented high Miller-index domains (field of view 520 µm); b) EBSD color-coded map of the same region with crystallographic orientations indicated by Miller indices. The inverse pole figure is shown for reference in the bottom right corner; c) Examples of different stepped structures, visible in the field of view of (a) and (b), illustrated by ball models; d) Magnified view of a 45 x 45 µm^2 region with a crystallographic $(\overline{25}\,\overline{5}\,38)$ orientation within the $(\overline{30}\,37\,\overline{6})$ domain. (a) and (b), illustrating the clean Rh sample, were in part adapted from Ref. [2] (Copyright © 2016, Springer Nature).

Stepped walls of the furrow-like defect on Rh(111)

The confined oscillations in H_2 oxidation were observed within a mesoscopic furrow-like defect (a surface indentation, 25-30 µm wide, 1 µm deep; a "scratch", Supplementary Figure 2a) on the smooth Rh(111) single crystal surface.

The atomic force microscope (AFM) and EBSD measurements confirm the presence of highly stepped surfaces on the walls of the furrow: Supplementary Figure 2b presents an EBSD color-coded map with the Miller-indices of the wall planes indicated (which vary slightly along the furrow, remaining, however, always high Miller-index planes). Supplementary Figure 2c shows the AFM-measured profile of the furrow, illustrating the morphology variations along the furrow.

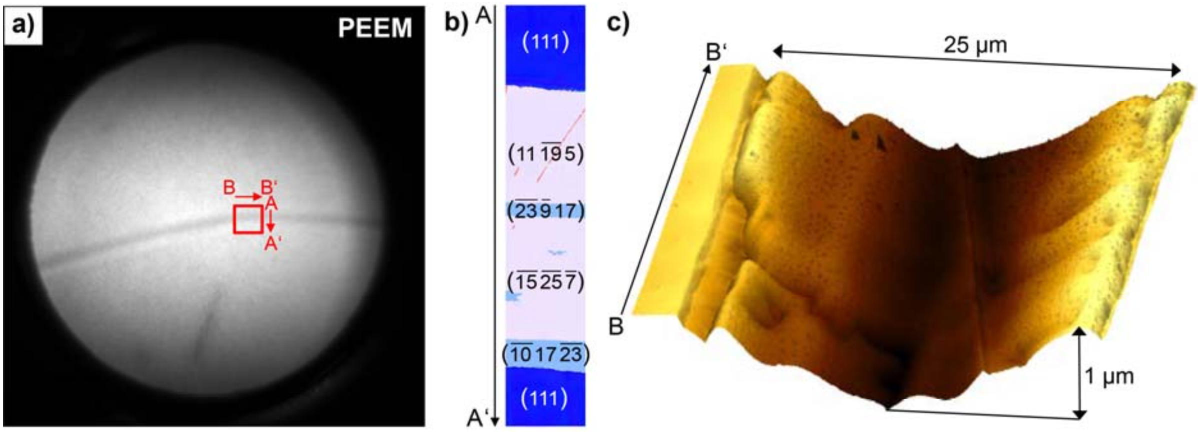

Supplementary Figure 2. Stepped walls of the furrow-like defect on Rh(111):
a) PEEM image (field of view 520 µm) of the Rh(111) surface containing a furrow-like defect (indentation), the red rectangle marks the region where the EBSD and AFM measurements were performed; b) EBSD color-coded map (7×35 µm^2) of a typical region of the furrow with the Miller-indices indicated. The color-codes correspond to the inverse pole figure in Supplementary Figure 1b; c) an example of the profile of the furrow, as imaged by AFM.

Supplementary Note 2: Oscillations propagating in space as chemical waves

Similar to the general understanding of the term *wave,* which implies a process with periodicity in space and time, a *chemical wave* consist of travelling concentration gradients of different species, which form periodical patters in space and exhibit temporal oscillations [3, 4]. In the present case, the travelling chemical waves consist of the concentration gradients of adsorbed hydrogen and oxygen, formed on the heterogeneous Rh foil surface. Since the surface work function depends on the adsorbate coverage, these waves can be directly visualized by PEEM.

Supplementary Figure 3a shows an illustration for heterogeneous Rh foil: both the periodicity in space and time can be revealed by analysis of PEEM video frames. The red curve in the inset of Supplementary Figure 3a shows the PEEM intensity variations along the x-axis marked in the rectangular area, the consecutive video-frames of the propagation of the blue-colored wave are shown in Figs.1c-f of the main text. The blue curve in the inset of Supplementary Figure 3a displays the time dependent PEEM intensity at ROI A.

Supplementary Figure 3b shows such dual periodicity for the furrow on the Rh(111) surface: the red curve shows the intensity variations in the red-marked region along the furrow, illustrating the spatial periodicity, the green curve (corresponding to the ROI 2 curve in Fig. 2b of the main text) demonstrates, in turn, the periodicity in time. The spatial periodicity along the furrow appears less perfect than for the domains of the Rh foil, since the density of steps varies along the furrow wall surface.

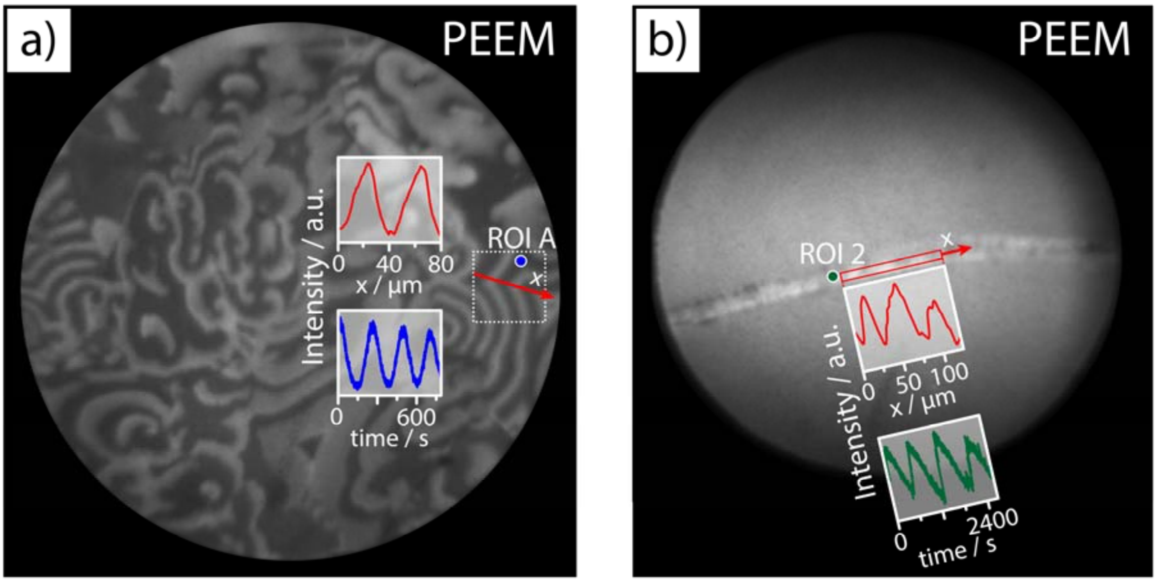

Supplementary Figure 3. Kinetic oscillations in H_2 oxidation, propagating as chemical waves on a Rh surface: a) PEEM image (field of view 520 µm), corresponding to Fig. 1a of the main text. The red curve illustrates the periodicity in space for the marked rectangular area corresponding to Fig. 1c of the main text and the blue curve demonstrates the periodicity in time measured in ROI A; b) the same, but for the furrow on the smooth Rh(111) surface (the PEEM image corresponds to Fig. 2a of the main text), the red curve illustrates the periodicity in space for the marked area placed along the furrow and the green curve demonstrates the periodicity in time measured in ROI 2. The PEEM intensity of the Rh(111) surface outside of the furrow remains constant (top and bottom curves in Fig. 2b of the main text).

Supplementary Note 3: Micro-kinetic model

The micro-kinetic model is based on the well-established Langmuir-Hinshelwood mechanism for H_2 oxidation on Rh [5], with the reaction network including the dissociative adsorption and associative desorption of hydrogen (R1), dissociative adsorption (and associative desorption) of oxygen via a precursor state (R2, R3), formation and reduction of subsurface oxygen (R4) and catalytic water formation [R5].

$$H_2 + 2* \leftrightarrow 2H* \qquad (R1)$$

$$O_2 \leftrightarrow O_2\,(pre) \qquad (R2)$$

$$O_2(pre) + 2* \leftrightarrow 2O* \qquad (R3)$$

$$O_* + s \leftrightarrow * + Os \qquad (R4)$$

$$2H* + O* \rightarrow H_2O + 3* \qquad (R5)$$

Here, * and s correspond to the empty surface and subsurface sites, respectively. In a mean-field approach, the partial coverages of hydrogen, oxygen and subsurface oxygen (θ_H, θ_O and θ_s, correspondingly) can be formulated by rate equations. In the present model, we assume that water is desorbed once it is formed. This is based on the fact that the simulations are performed for a temperature (433 K) which is above the desorption temperature of water on Rh(001) [6] being about 300 K. Readsorption of water is unlikely as the reactor (UHV PEEM chamber) is continuously pumped off.
To model the observed oscillations, we adapted the model developed by McEwen and co-authors describing field-induced oscillations in H_2 oxidation on Rh [7, 8], for a field free case. The resulting kinetic equations describing the mean site occupations for hydrogen, oxygen, and subsurface oxygen are:

$$\frac{d\theta_H}{dt} = 2k_a^H P_{H_2}\theta_*^2 - 2k_d^H\theta_H^2 - 2k_r\theta_H\theta_O$$

$$\frac{d\theta_O}{dt} = \frac{2}{1+K\theta_*^2}(k_a^O K P_{O_2}\theta_*^2 - k_d^O\theta_O^2) - k_{ox}\theta_O(1-\theta_s) + k_{red}\theta_s\theta_* - k_r\theta_H\theta_O$$

$$\frac{d\theta_s}{dt} = k_{ox}\theta_O(1-\theta_s) - k_{red}\theta_s\theta_*$$

The empty sites are given by $\theta_* = 1-\theta_H-\theta_O$ and the rate constants are given by:

$$k_A^H = S_0^H a_s / \sqrt{2\pi m_{H_2} k_B T}$$

$$k_a^O = S_0^O a_s / \sqrt{2\pi m_{O_2} k_B T}$$

$$k_d^H = k_{d0}^H e^{-\beta E_d^H}$$

$$K = K_0 e^{-\beta(E_K + A_K^O\theta_O + A_K^s\theta_s)}$$

$$k_d^O = k_{d0}^O e^{-\beta(E_d^O + A_d^O\theta_O + B_d^O\theta_O^2)}$$

$$k_{ox} = k_{ox}^0 e^{-\beta E_{ox}}$$

$$k_{red} = k_{red}^0 e^{-\beta(E_{red} + A_{red}^s\theta_s)}$$

$$k_r = k_r^0 e^{-\beta(E_r + A_r^H\theta_H + A_r^O\theta_O)}$$

The molecular masses are given by m_{H_2} and m_{O_2}. S_0^X denotes the initial sticking probability and a_s the area of a surface site (10 Å^2), $\beta = 1/k_B T$. The values for the kinetic parameters used in Figure 3b of the main text are listed in Supplementary Table 1. Apart from the activation energies for oxide formation and reduction and the sticking coefficient for hydrogen, the values in the Supplementary Table 1 correspond to the values previously determined for Rh(111) [8]. We note that the activation energies reported in Ref. [8] for oxide formation (E_{ox}) and oxide reduction (E_{red}) on Rh(111), Rh(011) and Rh(001) correlate linearly according to:

$$E_{red} = 0.293 + 0.776 E_{ox}$$

In the present simulations, E_{ox} was reduced until oscillations were obtained and E_{red} was scaled accordingly using the scaling relation. The corresponding E_{ox} value of 1.134 eV appeared to be lower than values of 1.68 eV used in Refs. [7-9] for Rh(111). We note, that the value of 1.68 eV was estimated in [9] by calculating diffusion of an O-atom trough the metal surface layer.

However, it is experimentally known that the oxidation of Rh(111) proceeds rather via steps than through the metal layer and that the rate depends on the direction of the oxidation [10]. In the present study, we are investigating highly stepped surfaces and it is, thus, reasonable that the barrier for oxidation is reduced with respect to the estimate in Ref. [9].

In Refs. [7, 8] the hydrogen sticking coefficient was set to be 0.3 in order to model the field-induced oscillations. We note that a slightly higher sticking coefficient (0.4) is required to obtain oscillations for the present field-free conditions, if the other parameters are kept constant.

Supplementary Table 1

E_d^H	0.70
S_0^O	0.60
A_K^s	0.082
E_{ox}	1.134
E_d^O	2.85
A_d^O	-0.4
B_d^O	-0.5
E_r	0.75
s_0^H	0.4
k_{d0}^H	3.0 x 10^{10}
K^0	0.2525
E_K	-0.178
A_K^O	0.158
k_{ox}^0	5.0 x 10^{11}
k_{red}^0	1.85 x10^{13}
A_{red}^s	0.3
k_{d0}^O	6.0 x 10^{13}
k_r	7.0 x 10^{12}
A_r^H	-0.27
A_r^O	-0.145

Calculation parameters for Rh(111): Energies are given in eV and rate constants in s^{-1}.

Supplementary References

[1] F.J Humphreys, *J. Mater. Sci.* **36** (2001) 3833

[2] M. Datler, I. Bespalov, S. Buhr, J. Zeininger, M. Stöger-Pollach, J. Bernardi, G. Rupprechter, and Y. Suchorski, *Catal. Lett.* **146** (2016) 1867

[3] A.N. Zaikin, A.M. Zhabotinsky, *Nature*, **225** (1970) 535

[4] S.K. Scott, *Oscillations, Waves and Chaos in Chemical Kinetics*, Oxford University Press, Oxford, U.K., 1995.

[5] M. P. Zum Mallen, W. R. Williams and L. D. Schmidt J. Phys. Chem. **97** (1993) 625 and references therein

[6] L. Gregoratti, A. Baraldi, V.R. Dhanak, G. Comelli, M. Kiskinova, R. Rosei, *Surf. Sci.* **340** (1995) 205

[7] J.-S. McEwen, P.Gaspard, T. Visart de Bocarme and N. Kruse, *Proc. Natl. Acad. Sci.* **106**, (2009) 3006 and SI therein

[8] J.-S. McEwen, P.Gaspard, T. Visart de Bocarme and N. Kruse, J. Phys. Chem. C **113** (2009) 17045.

[9] J.-S. McEwen, P.Gaspard, F. Mittendorfer, T. Visart de Bocarme and N. Kruse, Chem. Phys. Lett. **452** (2008) 133.

[10] J. Klikovits et al., Phys. Rev. Lett. **101** (2008) 266104.
