# Supplementary material for: Visualizing catalyst heterogeneity by a multifrequential oscillating reaction
Source: Nat Commun. 2018 Feb 9;9:600. doi: 10.1038/s41467-018-03007-3 (PMC5807506; doi:10.1038/s41467-018-03007-3)
Supplement: Supplementary file 2 — Description of Additional Supplementary Files [file 41467_2018_3007_MOESM2_ESM.pdf]

Description of Additional Supplementary Files

File Name: Supplementary Movie 1
Description: **In situ photoemission electron microscopy video**. Multifrequential oscillations occurring during the hydrogen oxidation reaction over a heterogeneous rhodium surface composed of µm-sized domains of different crystallographic orientations. The video reveals a complex turbulence-like “stirring” surface consisting of repeatedly nucleating "spirals" which spread as chemical waves.
